# Disseminated Cryptococcosis With Prostate Involvement in a Patient With T-cell Prolymphocytic Leukemia and Prostate Cancer

**DOI:** 10.7759/cureus.61555

**Published:** 2024-06-02

**Authors:** Nasos Ioannidis, Charalampos Mavridis, Georgios Anagnostakis, Georgia Tsoumi, Stamatis Karakonstantis, Irene Xylouri, Elias Drakos, Charalampos Mamoulakis

**Affiliations:** 1 Urology Department, University General Hospital of Heraklion, University of Crete Medical School, Heraklion, GRC; 2 Hematology Department, University General Hospital of Heraklion, University of Crete Medical School, Heraklion, GRC; 3 Internal Medicine Department, University General Hospital of Heraklion, University of Crete Medical School, Heraklion, GRC; 4 Pathology Department, University General Hospital of Heraklion, University of Crete Medical School, Heraklion, GRC

**Keywords:** allogeneic transplantation, cryptococcus, prostate cancer, stem cell transplantation, t-cell prolymphocytic leukemia

## Abstract

T-cell prolymphocytic leukemia (T-PLL) presents unique treatment challenges because of its rarity and aggressiveness. Allogeneic hematopoietic stem cell transplantation offers a potentially curative option, but its safety in patients with concurrent invasive fungal infections and solid malignancies remains uncertain. We present a case of a 68-year-old male with T-PLL who developed disseminated cryptococcal disease with prostate involvement and concurrent prostate cancer (PCa). Despite the challenges, successful control of the infection and radical prostatectomy enabled the patient to proceed safely to allogeneic transplantation. The case highlights the importance of vigilance for unusual infections, such as Cryptococcus, in immunocompromised patients presenting with lower urinary tract symptoms. Clinicians should consider the possibility of PCa in this population, particularly in the context of chronic leukemia. Concurrently, the potential association between fungal prostate infections and PCa warrants further investigation.

## Introduction

T-cell prolymphocytic leukemia (T-PLL) is a rare and aggressive type of hematological malignancy characterized by the abnormal proliferation of mature T lymphocytes in the blood, bone marrow, and various organs [1]. The treatment options for T-PLL are limited, and alemtuzumab remains the first-line therapy for treatment-naive and relapsed/refractory patients [2]. Other agents commonly used in treating T-PLL include fludarabine and cyclophosphamide [2]. Allogeneic hematopoietic stem cell transplantation may be considered for eligible patients (30-50% of the total), particularly for those who achieve a response to initial therapy, which may lead to rare-curative outcomes and superior disease control [2]. However, the literature on the timing and safety of transplantation in patients with invasive fungal infections is limited [3-5]. A further concern is the safety of transplantation in patients with a history of solid malignancy [6].

Cryptococcosis is a fungal infection caused mainly by Cryptococcus neoformans and Cryptococcus gattii [7]. It is an opportunistic infection affecting immunocompromised or immunosuppressed people who become infected by inhaling fungal spores present in the environment (soil contaminated with bird droppings) [7]. Except for the lungs, Cryptococcus can hematogenously spread to other parts of the body, mainly the central nervous system and the skin [8]. The involvement of the prostate is rare [8].

In this report, we are presenting the case of a patient with T-PLL with disseminated cryptococcal disease, prostate involvement, and concurrent prostate cancer (PCa).

## Case presentation

**Figure 1:**
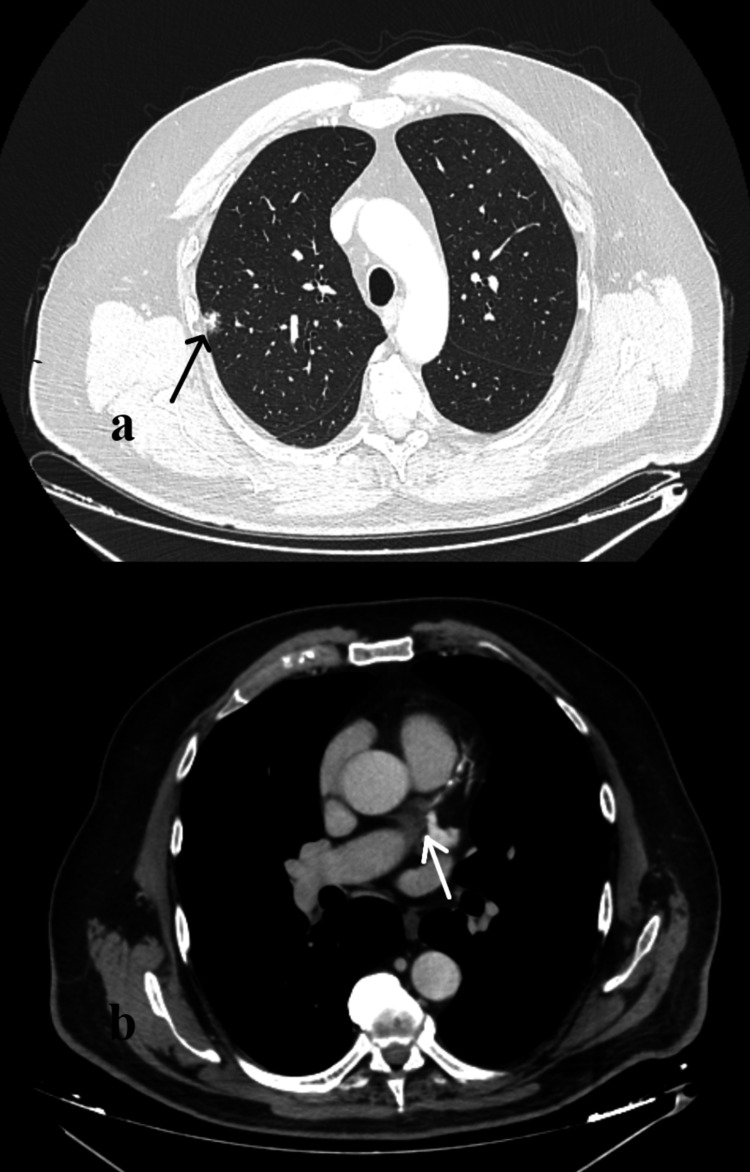
Chest CT (axial view) in the patient with T-PPL (a,b) (a) The black arrow shows a nodule with a halo sign. (b) The white arrow shows mediastinal lymphadenopathy.

A 68-year-old patient with a history of T-PLL (diagnosed with bone marrow biopsy and immune-phenotype a year before) and benign prostate hyperplasia presented to the emergency department because of a high fever (39°C) and malaise. The patient was on maintenance therapy with alemtuzumab, administered monthly, starting eight months ago (following induction chemotherapy with four cycles of fludarabine, mitoxantrone, and cyclophosphamide, which was initiated at the time of diagnosis and completed three months later) and was scheduled for allogeneic stem cell transplantation. Blood cultures returned positive for Cryptococcus neoformans, and induction therapy with liposomal amphotericin B 4 mg/kg and fluconazole 800 mg daily (flucytosine unavailable) was initiated. Serum cryptococcal antigen was 1:1,024, while the cerebrospinal fluid examination was normal. A chest CT revealed two nodules (1.8 cm and 0.6 cm) with a halo sign and new mediastinal lymphadenopathy (Figure 1). The brain CT was normal.

The patient responded to treatment (defervescence and improvement of c-reactive protein (CRP). However, while still on induction therapy, the patient developed lower urinary tract symptoms (LUTS) with sterile urine culture, and CRP had plateaued at 7-8 mg/dL. The fluconazole dose was increased to 1,200 mg once daily, leading to further improved CRP (2 mg/dL). Serum prostate-specific antigen (PSA) was high (26 ng/mL), whereas one year ago, it was 3.5 ng/mL; hence, a transrectal ultrasound (TRUS) was performed. Multiple small abscesses were observed, especially in the right lobe, and two of them with a maximum diameter of less than one cm (0.8 and 0.9 cm, respectively) were drained with an 18-gauge needle under TRUS guidance. The paracentesis material was purulent, and the cultures came back positive for Cryptococcus neoformans, Enterococcus faecalis, and Escherichia coli. Ciprofloxacin 400 mg twice daily and amoxicillin 1 g thrice daily was initiated. Liposomal amphotericin B induction was stopped after about three weeks of treatment, considering clinical improvement, and the patients remained on fluconazole consolidation therapy. Symptoms improved, and a follow-up TRUS showed the resolution of abscesses.

**Figure 2:**
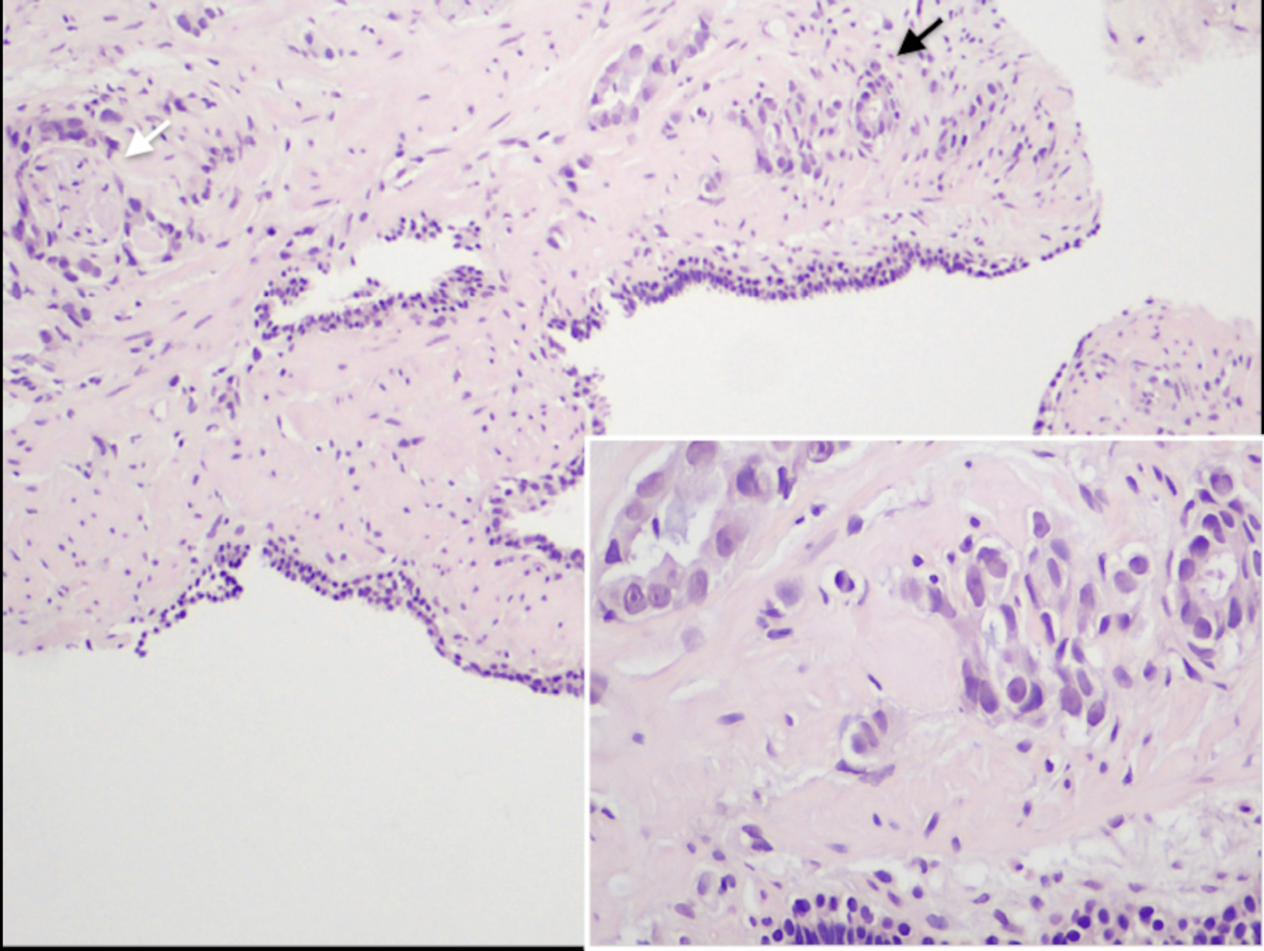
Histological findings of prostate cancer A prostate core biopsy disclosed focal lobular prostate adenocarcinoma (black arrow) with sites of perineural infiltration (white arrow). The primary Gleason pattern, shown at higher magnification in the inset, was 3, while focal areas with Gleason pattern 4 were also observed. Eosin and hematoxylin stains, with original magnification x100 (insets x400).

Histological samples were sent for assay as PSA values dropped to 12 ng/mL, while his prostate volume was 75 mL (PSA-density 0.16 ng/mL^2^). All symptoms, including respiratory and LUTS, had resolved. Histology from TRUS biopsy came back positive for PCa in two tissue samples from the right lobe with a Gleason score of 3 (primary) + 4 (secondary) and perineurial invasions (Figure 2).

**Figure 3:**
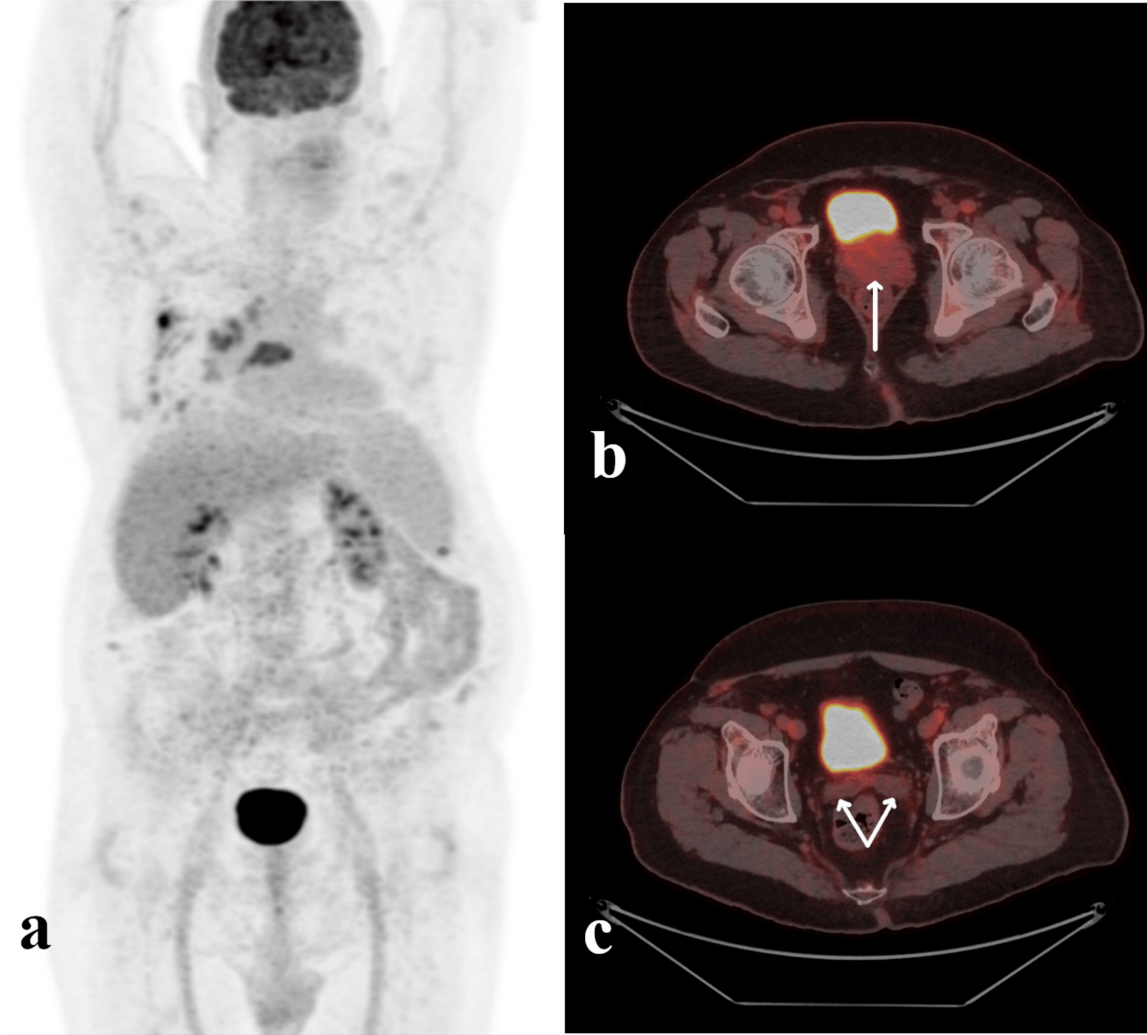
PET/CT imaging prior to radical prostatectomy (a,b,c) (a) PET scan, whole-body, coronal view. (b) PET/CT fusion images, axial view. The white arrow shows the prostate gland. (c) PET/CT fusion images, axial view. The double white arrows show the seminal vesicles.

The patient was discharged on fluconazole (to complete eight weeks of consolidation, followed by maintenance until planned allogeneic transplantation) and amoxicillin and ciprofloxacin (to complete six weeks of treatment). Before active treatment, the patient underwent a whole-body 18F-choline positron emission tomography (PET)/CT and abdominal contrast-enhanced CT. Unfortunately, prostate-specific membrane antigen (PSMA)/PET and magnetic resonance imaging (MRI) were unavailable. The imaging modalities did not detect distant metastases or pelvic lymph nodes (N0M0) (Figure 3).

An uncomplicated open radical prostatectomy without lymph node dissection was performed a month later, showing an International Society of Urological Pathology (ISUP) grade 2 (Gleason score 3+4), pT3a, and PCa in 2% of the right lobe and 1% of the left lobe. Considering sufficient control of the cryptococcal infection, the patient was referred for allogeneic transplantation, where, seven months later, he remained stable and PCa-free. The reported laboratory results during hospitalization and after the patient's discharge are presented in Table 1.

**Table 1:** Reported laboratory test results CRP, c-reactive protein; CSF, cerebrospinal fluid; DH, day of hospitalization; PSA, prostate-specific antigen; ED, emergency department; DD, discharge date; n/a, not applicable

Laboratory test	Reference range (units)	ED	DH4	DH5	DH11	DH18	DH31	DH51-DD	Nine months after DD
CRP	<0.5 (mg/dL)	5.8	16.8	14.3	8	7.9	3.4	2	1.2
Cryptococcal titers (CSF)	n/a	n/a	n/a	n/a	negative	n/a	n/a	n/a	n/a
Cryptococcal titers (serum)	n/a	n/a	n/a	1:1,024	n/a	n/a	n/a	n/a	n/a
PSA	<4 (ng/mL)	n/a	n/a	n/a	n/a	26	12	n/a	0

## Discussion

Cryptococcal prostatitis is a rare but recognized complication of disseminated cryptococcal infection, mainly in immunodeficient patients [8]. The available literature is limited to case reports or small case series [7-9]. Medical treatment may be sufficient, but prostatectomy may sometimes be necessary to eradicate the infection. To the best of our knowledge, the coexistence of PCa with cryptococcal prostatitis has only been described in a single patient [8].

It is well-established that prostate inflammation predisposes to the development of cancer through a long-term process [10]. However, data on the role of fungi are scarce. A distinct circulating fungal microbiome (including a higher frequency of Cryptococcus ater) has been reported in PCa patients compared to healthy individuals [10]. Additionally, Diniz-Lima et al., in their review, examined whether polysaccharides from Cryptococcus could contribute to carcinogenesis via their immunomodulatory and cytotoxic properties [11]. Moreover, genetic alterations in TCL1a proto-oncogene could be observed in both T-PLL and PCa, suggesting possible common pathways [12]. These data support the biological hypothesis of an association of Cryptococcus in the prostate with the development of PCa.

A difficult issue in the management of this patient was the appropriate timing and feasibility of hematopoietic stem cell transplantation considering the underlying cryptococcal infection and the underlying PCa. The literature on the topic is limited to small case series, but experience suggests that transplantation is safe if the fungal infection is well-controlled [3-5]. Furthermore, hematopoietic stem cell transplantation appears to be safe in patients with PCa in remission [6]. The guidelines of the European Association of Urology (EAU) and the American Urological Association (AUA) recommend surgical treatment in patients with a life expectancy of at least ten years [13]. However, we decided to treat the patient with a radical prostatectomy. The patient was being scheduled for allogeneic transplantation. Therefore, it was essential to manage any active malignancy before that. Additionally, there would be better management of prostatic cryptococcosis concerning a future relapse. Finally, Vardell et al. reported that 10%-20% of patients with T-PLL will survive ten years, especially those transplanted [14]. Interestingly, the surgery had no technical difficulties because of prostatic cryptococcosis, and the patient recovered promptly.

In our case, prostatectomy served two purposes: (1) eradication of this difficult-to-treat site of infection and (2) removal of the malignancy. Considering the sufficient control of the fungal infection and the PCa, the patient could safely proceed to stem cell transplantation.

## Conclusions

An immunocompromised patient with LUTS should be screened for possible prostate abscesses while obtaining samples for culture. Additionally, clinicians should consider the possibility of the presence of PCa in these patient groups. Further studies should investigate the possible association of fungal prostate infections with PCa, especially in patients with a history of chronic leukemia.

## References

[1] Colon Ramos A, Tarekegn K, Aujla A, Garcia de de Jesus K, Gupta S (2021). T-cell prolymphocytic leukemia: an overview of current and future approaches. Cureus.

[2] Staber PB, Herling M, Bellido M (2019). Consensus criteria for diagnosis, staging, and treatment response assessment of T-cell prolymphocytic leukemia. Blood.

[3] Puerta-Alcalde P, Champlin RE, Kontoyiannis DP (2020). How I perform hematopoietic stem cell transplantation on patients with a history of invasive fungal disease. Blood.

[4] Kashima E, Nagaharu K, Ino K, Sugimoto Y, Fujieda A, Kawakami K, Tawara I (2021). Voriconazole as a secondary prophylaxis for cryptococcal meningitis during hematopoietic stem cell transplantation. IDCases.

[5] Cahuayme-Zuniga L, Kontoyiannis DP (2010). Is it safe to proceed with stem cell transplant in cancer patients treated for cryptococcal infection? A focus on recent IDSA cryptococcal guidelines. Clin Infect Dis.

[6] Palmieri R, Montgomery RB, Doney K (2023). Allogeneic stem cell transplantation in patients with a prior history of prostate cancer. Ann Hematol.

[7] Baomo L, Guofen Z, Jie D, Liu X, Shuru C, Jing L (2024). Disseminated cryptococcosis in a patient with idiopathic CD4 + T lymphocytopenia presenting as prostate and adrenal nodules: diagnosis from pathology and mNGS, a case report. BMC Infect Dis.

[8] Shah SI, Bui H, Velasco N, Rungta S (2017). Incidental finding of cryptococcus on prostate biopsy for prostate adenocarcinoma following cardiac transplant: case report and review of the literature. Am J Case Rep.

[9] Xu L, Tao R, Zhao Q, Cheng J, Zhu B (2019). An AIDS patient with urine retention. BMC Infect Dis.

[10] Wang X, Zhou Z, Turner D, Lilly M, Ou T, Jiang W (2022). Differential circulating fungal microbiome in prostate cancer patients compared to healthy control individuals. J Immunol Res.

[11] Diniz-Lima I, da Fonseca LM, Dos Reis JS (2023). Non-self glycan structures as possible modulators of cancer progression: would polysaccharides from Cryptococcus spp. impact this phenomenon?. Braz J Microbiol.

[12] Sun S, Fang W (2020). Current understandings on T-cell prolymphocytic leukemia and its association with TCL1 proto-oncogene. Biomed Pharmacother.

[13] Ubrig B, Boy A, Heiland M, Roosen A (2018). Outcome of robotic radical prostatectomy in men over 74. J Endourol.

[14] Vardell VA, Ermann DA, Fitzgerald L, Shah H, Hu B, Stephens DM (2024). T-cell prolymphocytic leukemia: epidemiology and survival trends in the era of novel treatments. Am J Hematol.

